# Supplementary material for: Ultra-low power carbon nanotube/porphyrin synaptic arrays for persistent photoconductivity and neuromorphic computing
Source: Nat Commun. 2024 Jul 21;15:6147. doi: 10.1038/s41467-024-50490-y (PMC11271480; doi:10.1038/s41467-024-50490-y)
Supplement: Supplementary file 1 — Supplementary Information [file 41467_2024_50490_MOESM1_ESM.pdf]

Ultra-Low Power Carbon Nanotube/Porphyrin Synaptic Arrays for Persistent Photoconductivity and Neuromorphic Computing

Jian Yao, Qinan Wang, Yong Zhang, Yu Teng, Jing Li, Pin Zhao, Chun Zhao*, Ziyi Hu, Zongjie Shen, Liwei Liu, Dan Tian, Song Qiu, Zhongrui Wang, Lixing Kang*, Qingwen Li*

Authors and Affiliations

Jian Yao, Liwei Liu, Song Qiu, Lixing Kang, Qingwen Li

School of Nano-Tech and Nano-Bionics, University of Science and Technology of China, Hefei 230026, China

Jian Yao, Qinan Wang, Yong Zhang, Yu Teng, Jing Li, Pin Zhao, Ziyi Hu, Zongjie Shen, Liwei Liu, Song Qiu, Lixing Kang, Qingwen Li

Advanced Materials Division, Suzhou Institute of Nano-Tech and Nano-Bionics, Chinese Academy of Sciences, Suzhou 215123, China

Qinan Wang, Chun Zhao

School of Advanced Technology, Xi'an Jiaotong-Liverpool University, Suzhou 215123, China

Dan Tian

College of Materials Science and Engineering, Co-Innovation Center of Effiicient Processing and Utilization of Forest Resources, Nanjing Forestry University, Nanjing 210037, China

Zhongrui Wang

Department of Electrical and Electronic Engineering, University of Hong Kong, Pokfulam Road, Hong Kong SAR, China

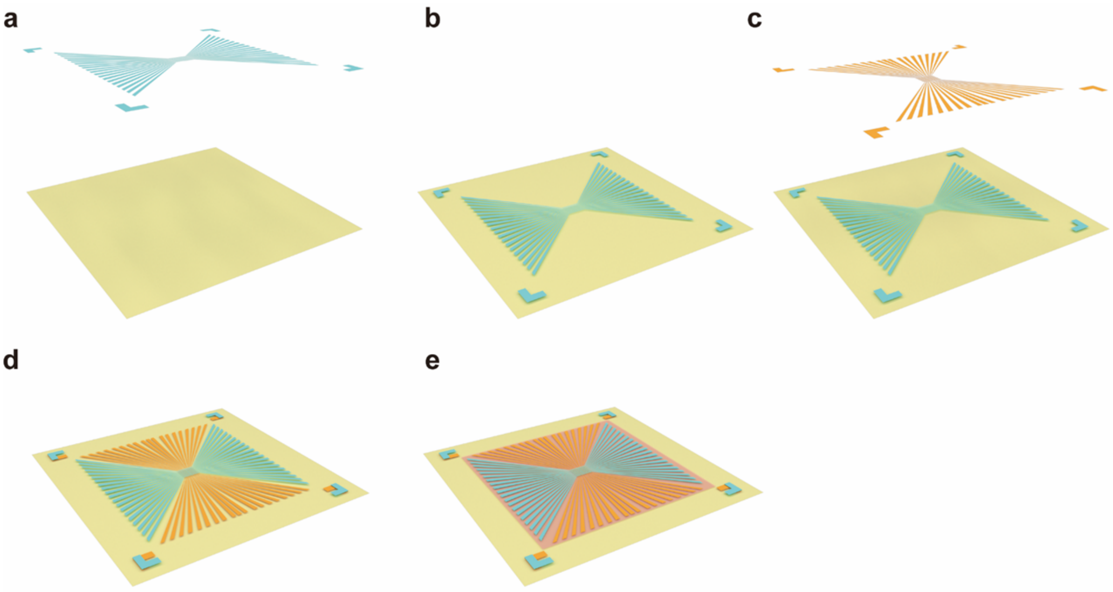

Supplementary Figure S1. Fabrication process for wafer-scale device arrays, illustrated using a single array as a representative example. (a) Gate electrode fabrication on a PI substrate (Ti/Au: 10/50 nm); (b) Atomic layer deposition (ALD) of a 70 nm HfO_2 layer at 250℃, followed by interconnect window opening using ion beam etching (IBE) technique, and subsequent deposition of a thin film of SWCNTs by solution-based methods, the PCz on the surface of SWCNTs film was cleaned with tetrahydrofuran; (c) Creation of source/drain electrodes and interconnecting lines (Ti/Au: 10/50 nm), involving channel definition and removal of excess SWCNTs thin film; (e) Spin-coating ZnTPP. The devices arrays can be easily detached from the silicon wafer using electrolytic delamination.[1,2]

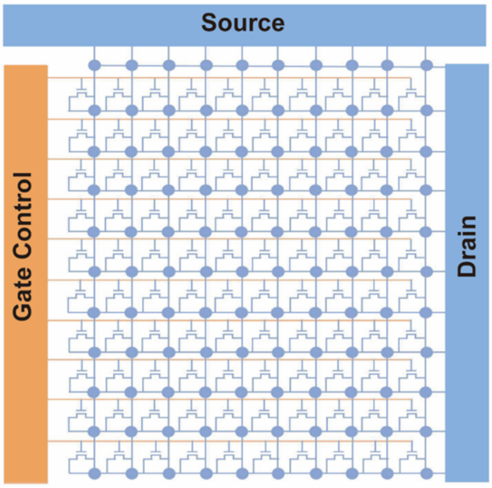

Supplementary Figure S2. 10×10 array circuit diagram, all 100 devices were tested on a flexible substrate using manual probe operations.

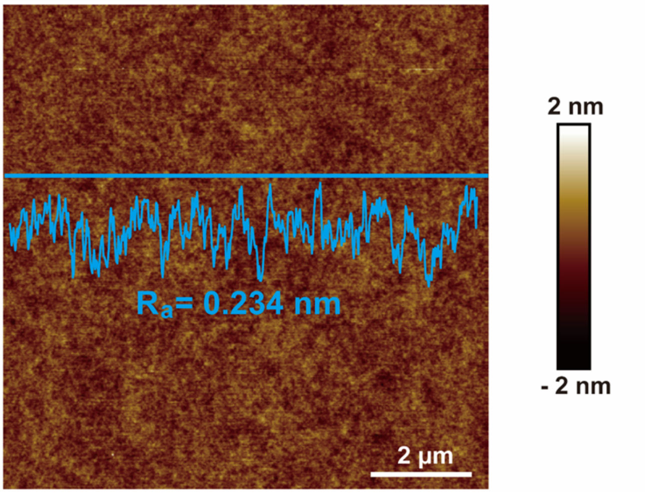

Supplementary Figure S3. The atomic force microscope (AFM) photograph of the spin coated PI (polyimide) film for the fabrication of SWCNTs/ ZnTPP device array, showing ultra-smooth surface with a contour arithmetic mean deviation (R_a) roughness less than 0.5 nm.

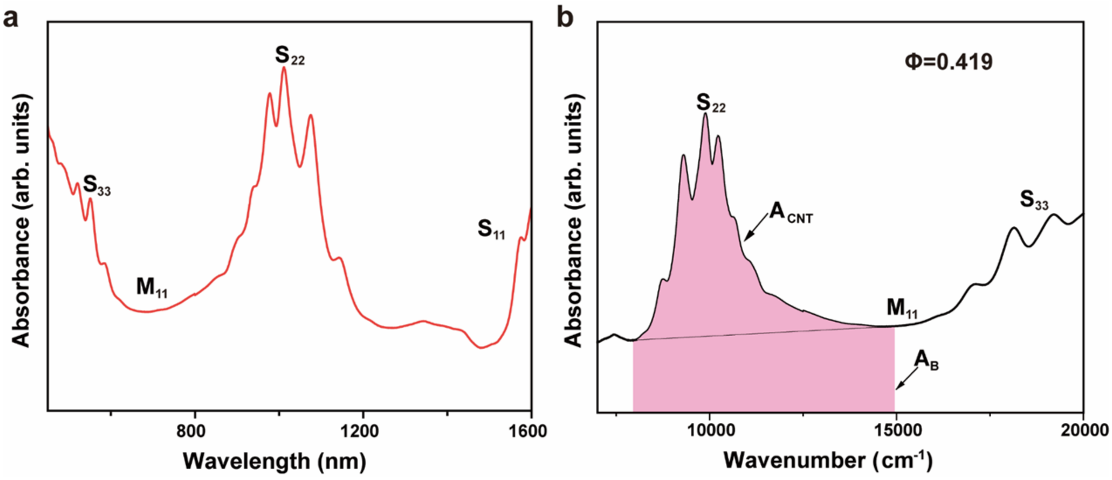

Supplementary Figure S4. Characterizing the purity of semiconducting-type carbon nanotubes through absorption spectrum. (a) The absorption spectra of SWCNTs solutions. (b) Absorption spectrum of the purified SWCNTs for calculating absorption peak ratio (ϕ).

In general, the semiconductor purity of SWCNTs solutions can be determined by absorption spectrum on a reported method. This method is applicable for the removal of the M_{11} peak due to the elimination of metallic CNTs by calculated absorption peak ratio (ϕ).

$$\phi_i = A_{\mathrm{CNT}}/(A_{\mathrm{CNT}} + A_{\mathrm{B}})$$

where A_{CNT} (red area) represents the enveloping area of the M_{11} and S_{22} bands enclosed by the linear baseline (dotted line), indicating the proportion of metallic single-wall carbon nanotubes (m-SWCNTs) and semiconducting single-wall carbon nanotubes (s-SWCNTs) in the sample, as shown in **Figure S4a**. Meanwhile, A_B denotes the area covered by the linear baseline in the same region, as shown in **Figure S4b** primarily associated with the presence of amorphous carbon impurities. The purity of s-SWCNTs in our study exceeds 99.99%, as evidenced by ϕ (0.419) being greater than the value (0.404) reported in a previous study.[3]

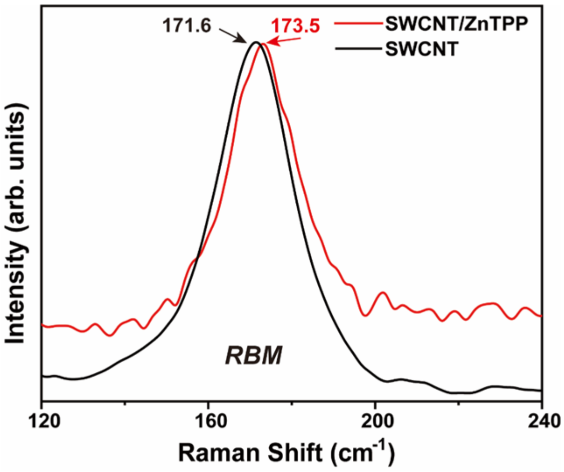

Supplementary Figure S5. The RBM region of SWCNTs and SWCNT/ ZnTPP on a silicon oxide substrate.

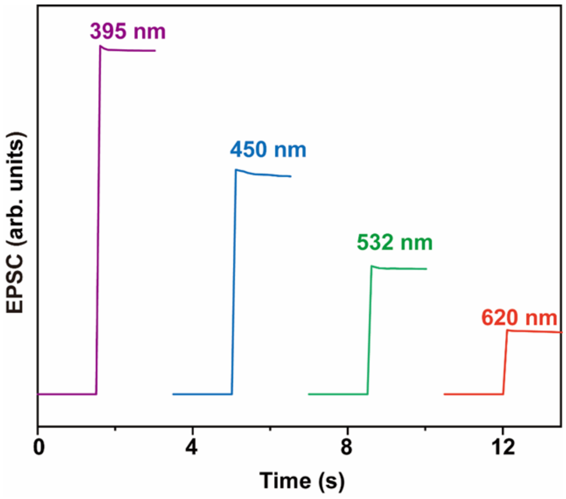

Supplementary Figure S6. The photocurrent generated by the device under different wavelength illumination conditions. The light pulse has a width of 0.1s, P_{light}=0.5 mW/cm^{-2}, V_{DS} = 1V

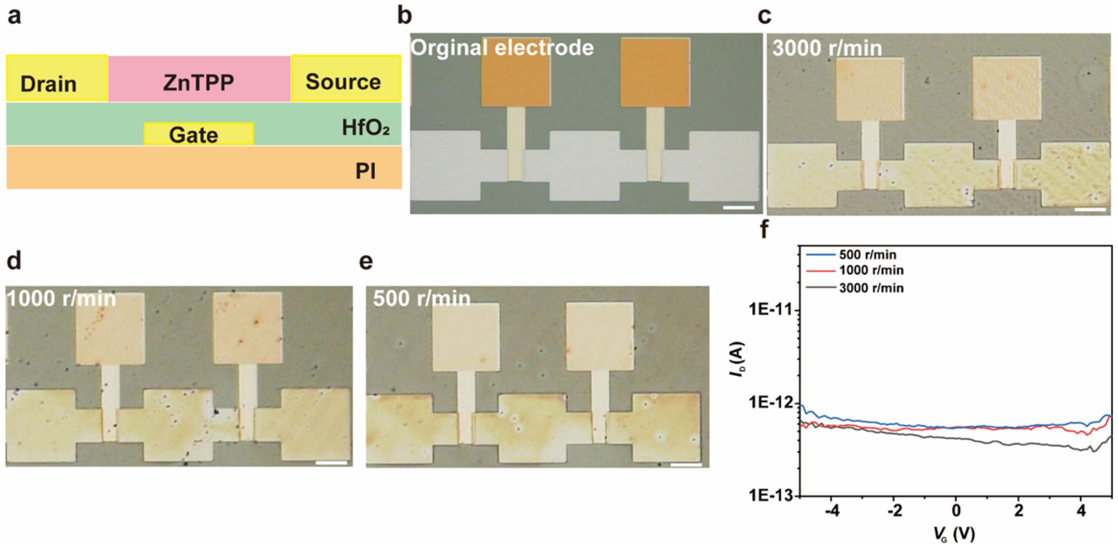

Supplementary Figure S7. (a) The diagram of device structure. (b), (c), (d), (e) Optical image of device after ZnTPP spin coating, the spin coating conditions are 500 r/min, 1000 r/min, 3000 r/min, respectively. The spinning time was 30 s. (scale bar: 40 μm) (f) Transfer characteristic curves of devices at different spin coating speeds. (V_{DS}=1 V)

Because ZnTPP can be dissolved in other organic solvents such as acetone, we structured the device with a bottom-gate bottom-contact (as shown in Figure **S7a**) configuration to mitigate the impact on ZnTPP during device fabrication. The specific process involved initially preparing the bottom gate electrode on the PI substrate through photolithography, followed by ALD growth of a 50 nm HfO_2 as the dielectric layer. Subsequently, the source and drain electrode was prepared by photolithography process (W=40 μm, L=20 μm). Finally, we coated the ZnTPP solution onto the prepared substrate surface using spin coating. As shown in **Figure S7b**, **S7c**, **S7d**, **S7e**, respectively. To ensure experimental accuracy, we implemented three different spinning conditions: 500r/min, 1000r/min, and 3000r/min, each for 30 s. Electrical measurements were conducted on the devices at room temperature. As shown in **Figure S7f**, the devices were unable to conduct under all three conditions, indicating that the ZnTPP thin films prepared by spin coating were inadequate as channel

layer materials due to their inability to form continuous and dense films. This also demonstrates that ZnTPP primarily functions as a photosensitive layer material in this work.

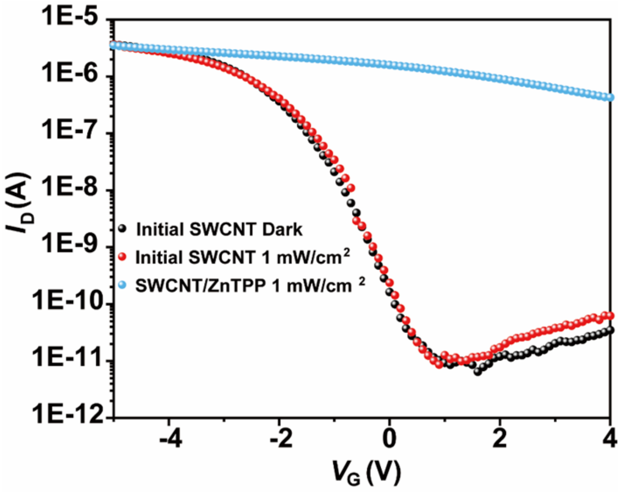

Supplementary Figure S8. Transfer curves of the device after spin-coating ZnTPP under dark and illumination conditions, respectively. (V_{DS} = 1 V, λ = 395 nm)

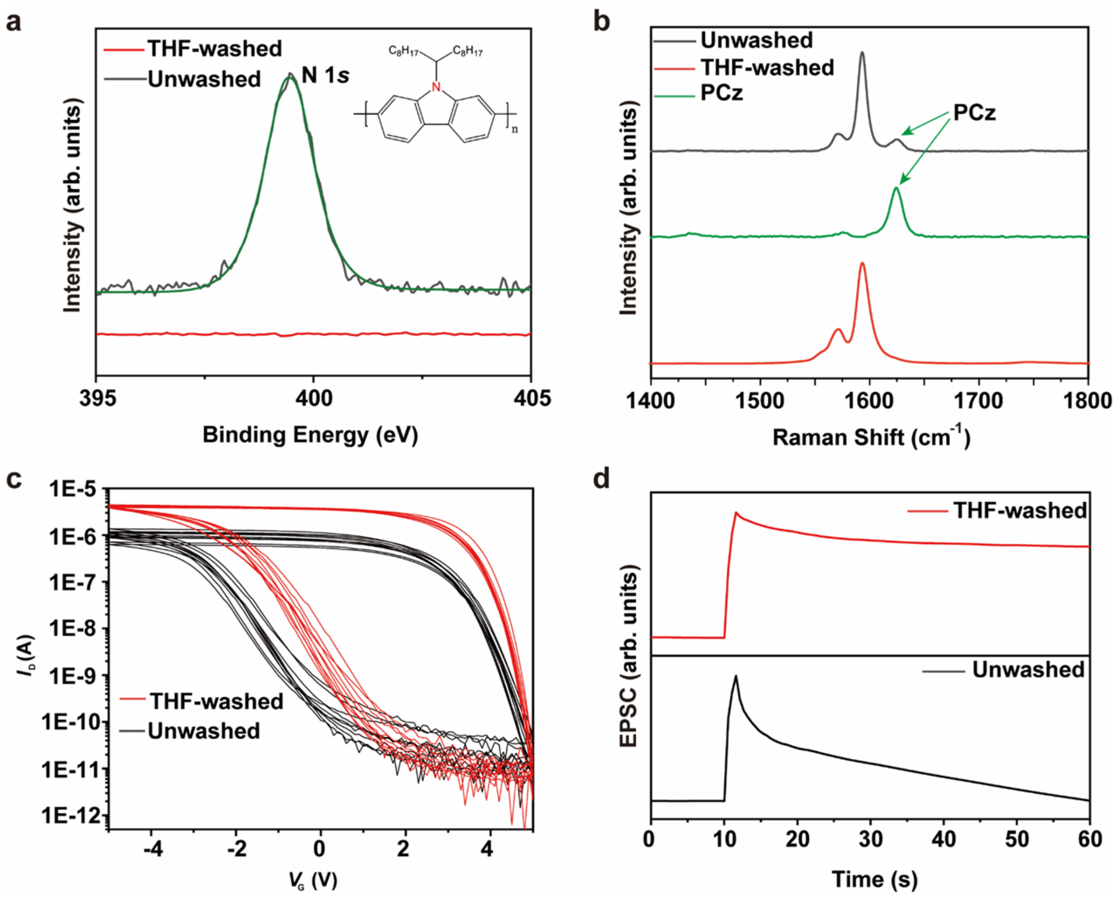

Figure S9. (a) The XPS results of surface N element on SWCNT thin films before and after THF washed. (b) The Raman results of pure PCz, and SWCNT thin films before and after THF washed. (c) The transfer characteristic curves of 10 SWCNT thin-film transistors with THF-washed and unwashed 10 SWCNT thin-film transistors unwashed were measured under dark, room temperature conditions. (d) The retention characteristics of the transistor with THF-washed and that unwashed were measured separately. (P_{light}=0.5 mW/cm^{-2}, V_{DS} = 1 V.)

Figure S9a illustrates the chemical structure of PCz. Initially, we conducted X-ray photoelectron spectroscopy (XPS) characterization of the films before and after THF washed. As depicted in **Figure S9a**, noticeable nitrogen elements were detected on the surface of films without THF washed, whereas no nitrogen elements were detected on THF-washed film surfaces, indicating the effective removal of PCz from SWCNTs surfaces during washing. Raman spectroscopy results further corroborated this conclusion. We measured pure PCz, films of SWCNTs without THF washed, and films after THF washed, as shown in **Figure S9b**. Pure PCz exhibited characteristic peaks around 1624 cm^{-1}. Typical PCz peaks were observed on films without THF

washed, while no PCz characteristic peaks were detected on THF-washed film surfaces, confirming the efficacy of THF washed in polymer removal. Additionally, examination of 100 sets of Raman results from **Figure S22** revealed no significant PCz signals, indicating uniform and thorough cleaning of the entire 4-inch wafer film.

Furthermore, using the same process, we fabricated transistor devices from films with and without THF washed, testing a total of 20 devices. As depicted in **Figure S9c**, the results indicated significantly lower on-state currents for devices without washed compared to those with washed, suggesting that the presence of PCz severely affects the direct contact between metal electrodes and SWCNTs, resulting in increased contact resistance and reduced on-state current. Devices subjected to THF washed exhibited a more uniform distribution of on-state currents compared to those unwashed, facilitating wafer manufacturing. It's important to note that this differs from the transfer characteristics curve provided in the supplementary information, as the testing here focused on pure SWCNTs, while the supplementary information presented SWCNT/ZnTPP transfer characteristics curves.

Subsequently, we spin-coated identical amounts of ZnTPP on device surfaces, evaluating their retention characteristics, as shown in **Figure S9d**. When subjected to the same pulsed light on both washed and unwashed devices, it was observed that the washed devices maintained excellent retention characteristics within the same test duration, whereas the unwashed devices exhibited almost no retention characteristics, with photocurrent decaying to the initial value within a short time. This indicates that the presence of PCz severely hinders direct contact between ZnTPP and SWCNT, making it difficult to form high-quality interfaces, thus indirectly demonstrating THF's excellent cleaning effect on PCz. The entire experimental outcome is predicated on the effective removal of PCz.

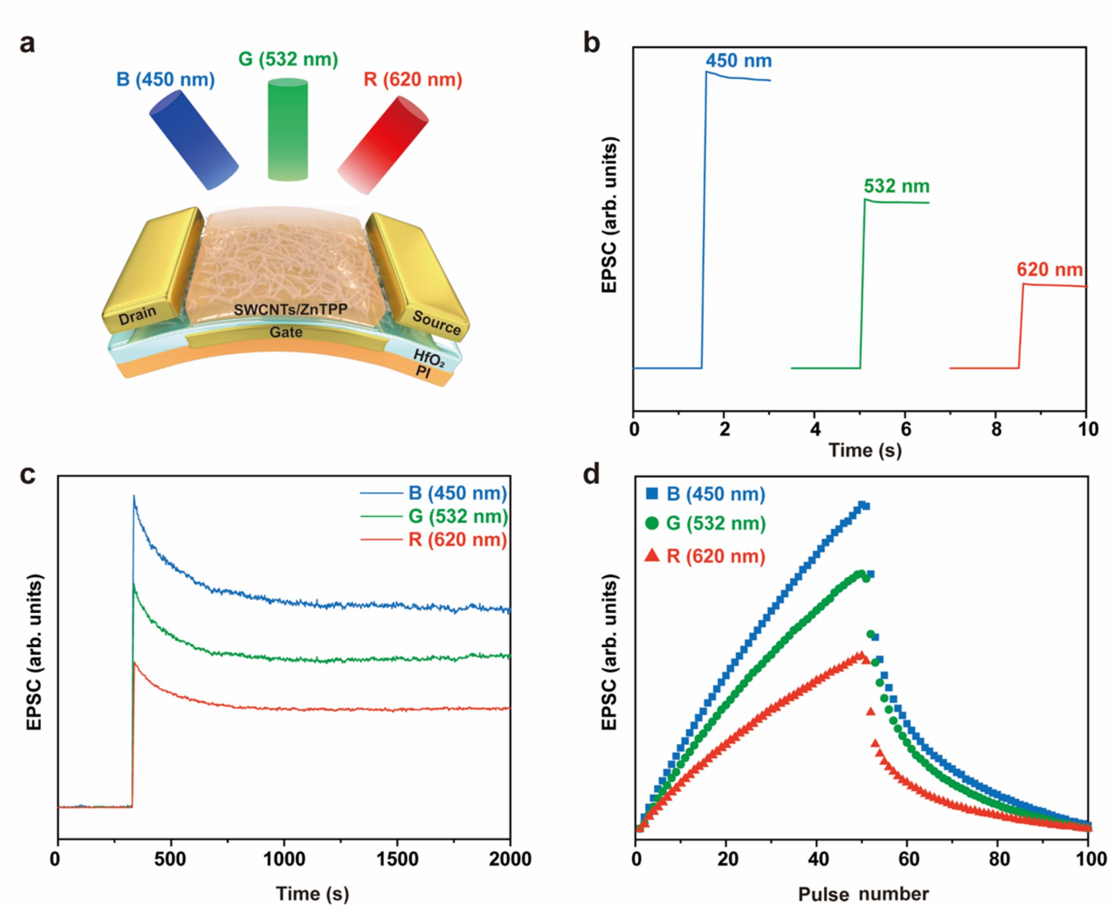

Supplementary Figure S10. (a) Schematic diagram of the SWCNTs and ZnTPP heterojunction device. (b) The photocurrent generated by the device under different wavelength illumination conditions. (P_{light}=0.5 mW/cm^{-2}, V_{DS} = 1 V), (c) Optical pulse intrigues a PPC state under 450 nm laser,532 nm laser,620 nm laser, respectively. (P_{light}=0.5 mW/cm^{-2} and V_{DS} = 1 V) (d) Light-controlled LTP (P_{light}=0.5 mW/cm^{-2}, duration of 1 s, spaced 2 s apart) and V_G-controlled LTD (V_G=−1.5 V, duration of 1 s, spaced 1s apart) for 50 pulses under 450 nm laser,532 nm laser,620 nm laser, respectively. V_{DS} = 1 V.

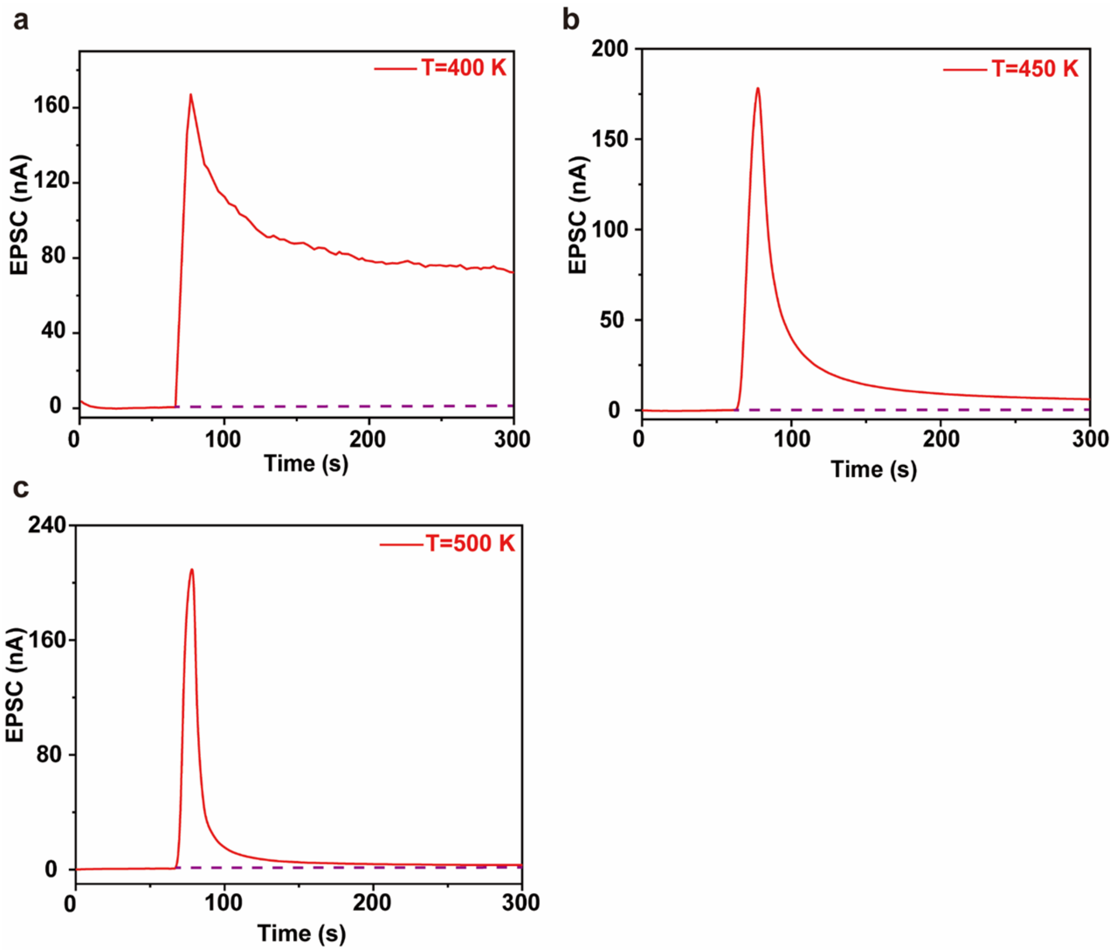

Supplementary Figure S11. The current storage capacity of the device after illumination at (a) 400 K, (b) 450 K, (c) 500 K, respectively. With the increase of temperature, the drift of electrons increases due to the disturbance of temperature, and the device gradually loses its storage capacity. The light pulse has a width of 10 s, P_{light}=0.5 mW/cm^{-2}, V_{DS} = 1 V.

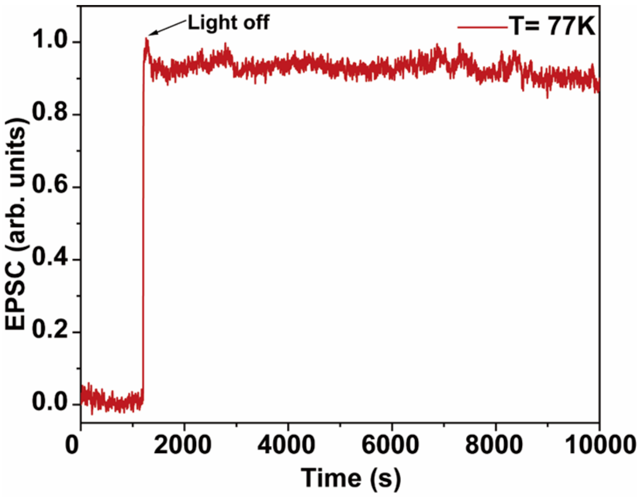

Supplementary Figure S12. Charge storage stability of the device, optical pulse intrigues a persistent photocurrent state in 77 K, low temperature can effectively suppress electron thermal motion, maintaining stable storage. The light pulse has a width of 30 s, P_{light}=0.5 mW/cm^{-2}, V_{DS} = 1 V.

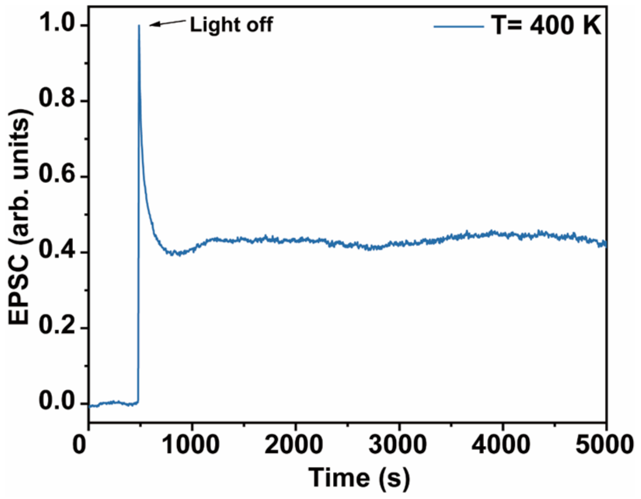

Supplementary Figure S13. Charge storage stability of the device, optical pulse intrigues a persistent photocurrent state in 400 K, high temperature intensifies electron thermal motion, leading to a decrease in storage capacity. The light pulse has a width of 30 s, P_{light}=0.5 mW/cm^{-2}, V_{DS}= 1 V.

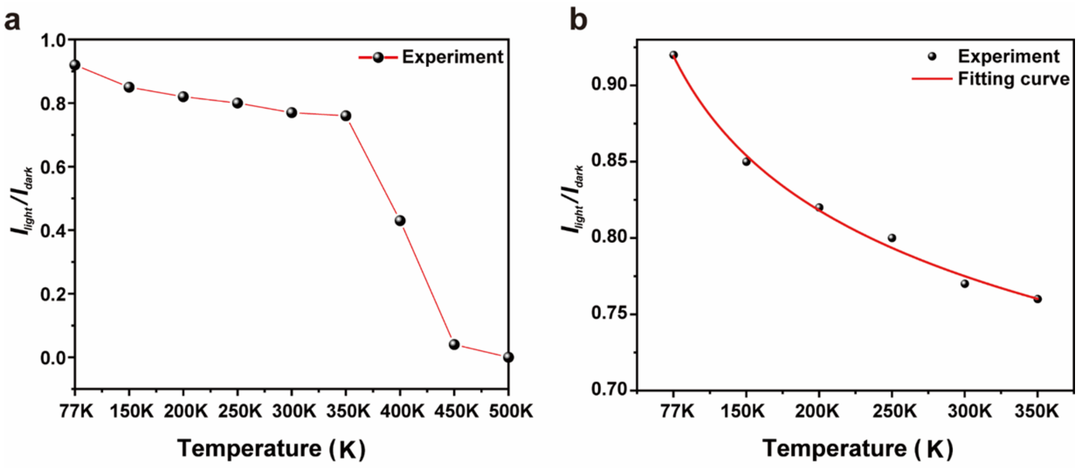

Supplementary Figure S14. (a) Comparison of storage capacities of the device at different temperatures, (b) In the temperature range from 77 K to 350 K, the relationship between temperature and η can be well fitted with a power-exponential function.

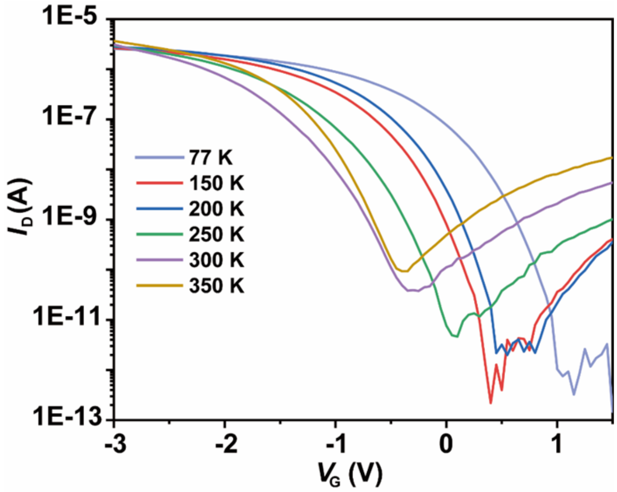

Supplementary Figure S15. Transfer characteristic curves of the device at different temperatures, $V_{DS} = 1$ V.

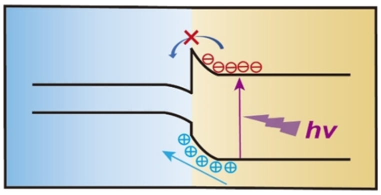

Supplementary Figure S16. Energy band structure of SWCNT and ZnTPP under illuminated conditions.

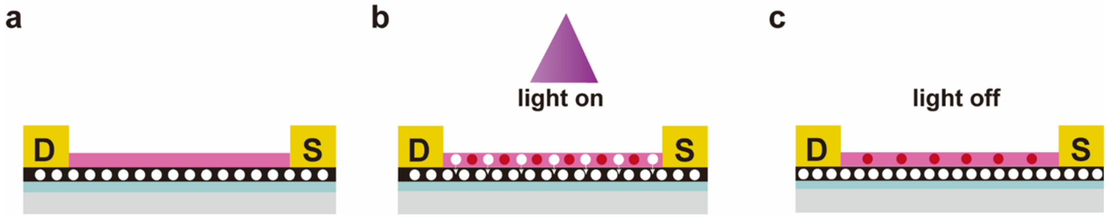

Supplementary Figure S17. The charge-trapping state of the device in dark condition, under light illumination and after the cessation of the illumination, respectively. Under dark conditions, SWCNTs exhibit P-type conductivity characteristics, resulting in the presence of numerous holes (white voids in **S17a**). When SWCNTs contact ZnTPP, a built-in electric field is formed due to energy band mismatch. Upon laser illumination, ZnTPP rapidly generates a multitude of hole-electron pairs due to the photoelectric effect. The electron-hole pairs swiftly separate under the influence of the internal electric field, with holes easily injected into SWCNTs while electrons remain in ZnTPP (red voids in **S17b**). The substantial potential barrier and defects at the interface prevents the recombination of these electrons, which is equivalent to applying a negative voltage to the surface of SWCNTs, inducing a persistent photocurrent in SWCNTs.

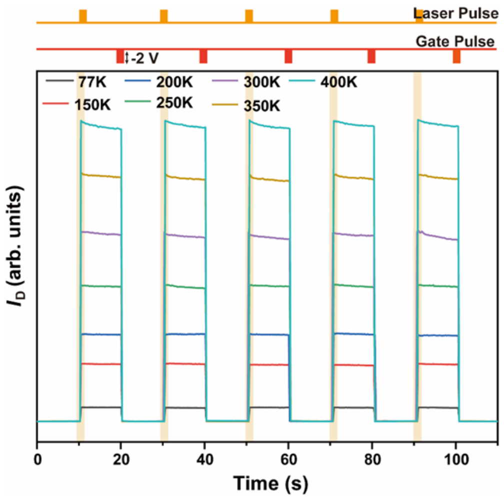

Supplementary Figure S18. The repeatability of light response at different temperatures. Using optical pulses for writing and electrical pulses for erasing. Both light pulse width and electrical pulses width are 0.2 s. (V_{DS} =0.1 V, P= 1 mW/cm^2)

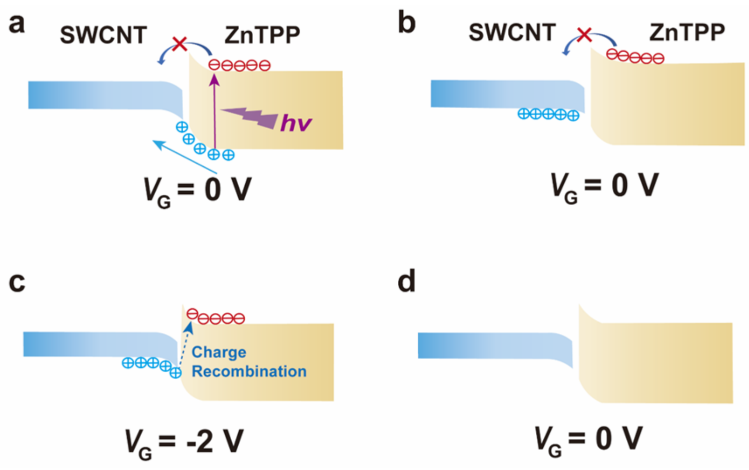

Supplementary Figure S19. Energy band gap diagram. (a) Energy band diagram under light illumination. (b) The electrons in ZnTPP continuously induce holes generation in SWCNTs. (c) Energy band diagram with -2 V gate pulse. (d) Energy band diagram after the pulse ends.

The band structure distribution of SWCNTs and ZnTPP in the initial state is depicted in **Figure 3g**. At this stage, due to the mismatched band structures of SWCNTs and ZnTPP, and the Fermi level of ZnTPP being higher than that of SWCNTs, ZnTPP transfers some electrons to SWCNT.[4–6] Meanwhile, the band of ZnTPP bends upwards, and that of SWCNTs bends downwards, resulting in a typical type I band structure. Upon illumination of the heterojunction, since ZnTPP is the main absorbing layer, numerous hole-electron pairs are rapidly generated in ZnTPP, as shown in **Figure S19a** (corresponding state I). Assisted by the built-in electric field, holes can easily transfer from ZnTPP to SWCNTs, leading to an increase in channel current. Meanwhile, due to the upward bending of the LUMO band of ZnTPP, which formed a certain barrier, hindering electrons recombination with holes. These electrons continue to be retained in ZnTPP. When the light is removed, these electrons continue to exist, thereby continuously inducing the generation of holes in SWCNTs, as shown in **Figure S19b** (corresponding state II), which is known as the photogating effect, which is the fundamental source of PPC in this work. The direction of electric field generated by the trapped electrons in the ZnTPP is opposite to the built-in field, so the degree of bandgap bending can be reduced.

When applied -2V gate pulse, the density of holes increases instantaneously, allowing the electrons previously blocked to recombine with the holes,[7,8] as shown in **Figure S19c** (corresponding state III). Upon pulse removal, the device returns to its initial state, as illustrated in **Figure S19d** (corresponding state IV).

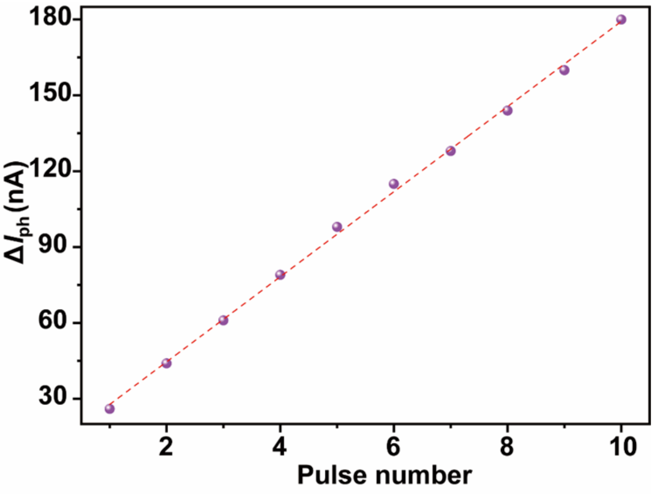

Supplementary Figure S20. The source-drain current varies with the number of pulses.

Table S1. Comparison of devices with persistent photoconductivity for latest literature studies

Active materials	Substrate	Gate Voltage (V)	Temperature (K)	Time (s)	Energy Per Spike (fJ)	Scale	Reference
SWCNT/ $FAPbBr_3$	Si/SiO_2	No	~50-RT	$\sim 5\times 10^3$	7.4	Single	Ref.9
BP/CdS	Si/SiO_2	No	RT	$> 5\times 10^3$	4.8	Single	Ref.10
MoS_2/PbS	Si/SiO_2	No	< 200	$> 10^4$	Not mentioned	Single	Ref.11
IGZO/PVK	PET/Al_2O_3	No	RT	$> 10^4$	Not mentioned	~2 cm ×2 cm	Ref.12
MoS_2/PO_x	Si/SiO_2	-60 V	80-300	$> 10^4$	Not mentioned	Single	Ref.13
Pentacene/ $CsPbBr_3$	Si/SiO_2	No	RT	$\sim 3\times 10^3$	~140	Single	Ref.14
VO_2	Si/SiO_2	No	RT	$\sim 4\times 10^3$	Not mentioned	2-inch	Ref.15
PDPP4T/ chlorophyll	Si/SiO_2	60 V	RT	$\sim 4\times 10^2$	0.25	Single	Ref.16
Graphene/ PQD	Si/SiO_2	10 V	RT	$\sim 3\times 10^3$	37000	Single	Ref.17
**SWCNT/ ZnTPP**	**PI/HfO_2**	**No**	**77-400**	**$> 2\times 10^4$**	**0.065**	**4-inch**	**This work**

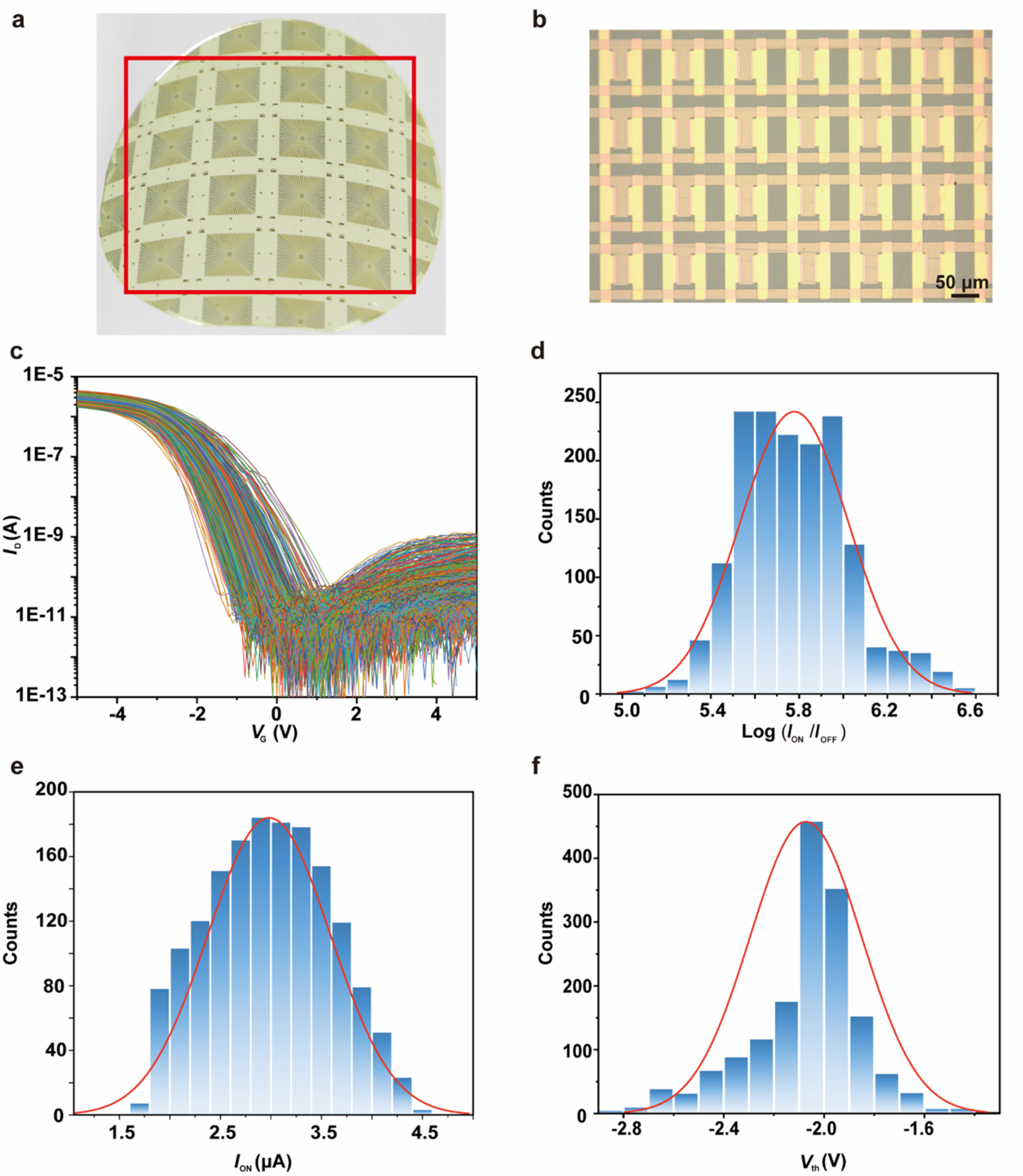

Supplementary Figure S21. (a) Optical photograph of a 4-inch flexible wafer arrays peeled off from the silicon substrate and (b) partial device from the array. (c) The transfer characteristic curves of 1600 SWCNT/ZnTPP phototransistors were measured under dark, room temperature conditions. These devices exhibited uniform electrical performance (V_{DS} = 1 V). (d) The statistical distribution of the on/off ratio. (e) The statistical distribution of on-state current. (f) The statistical distribution of threshold voltage (V_{th}). The red curve is an anormal curve.

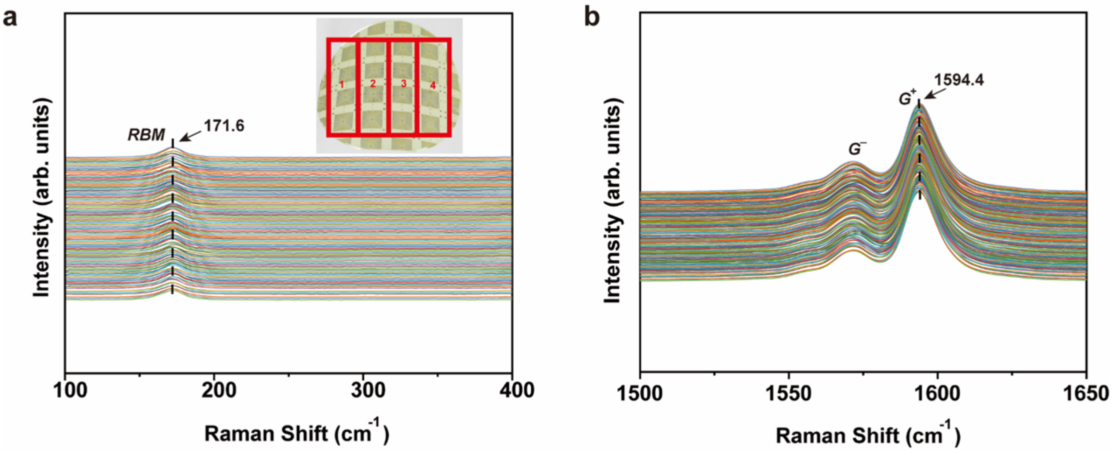

Supplementary Figure S22. Raman Characterization of 4-inch SWCNT film. (a) The *RBM* region of SWCNT films at 100 different positions. (b)The *G* region of carbon nanotube films at 100 different positions.

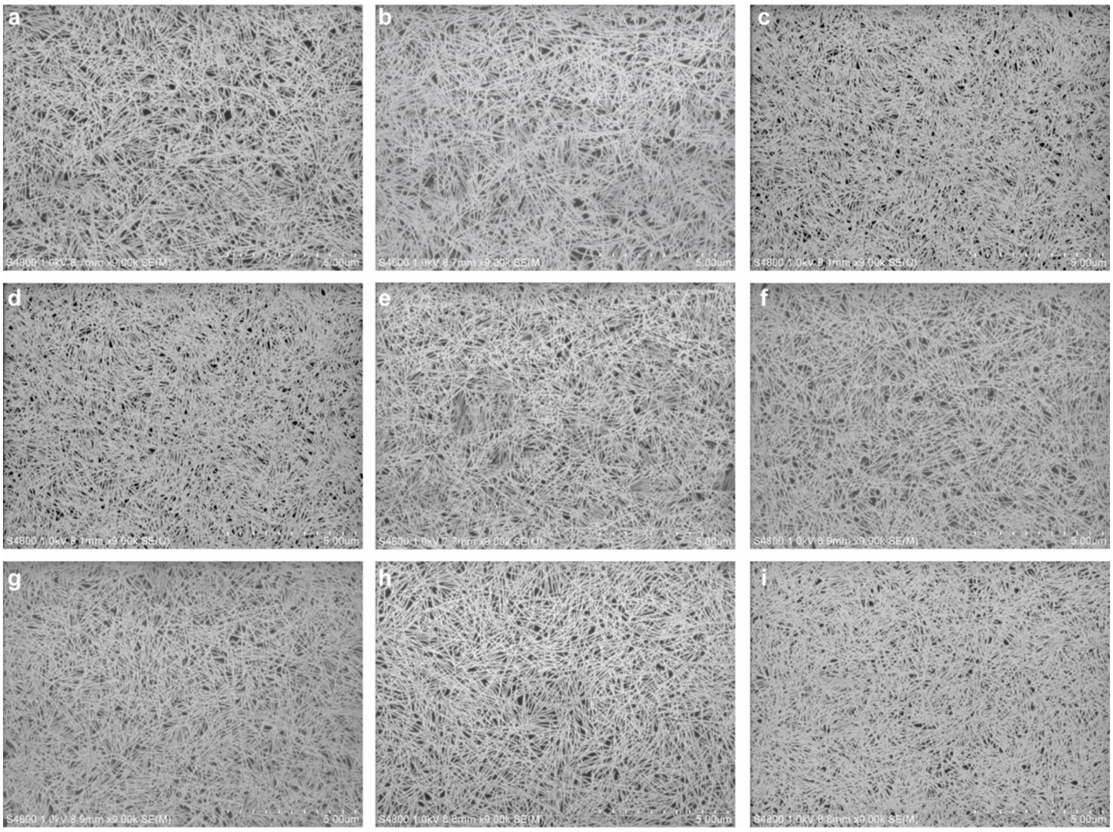

Supplementary Figure S23. SEM Characterization of SWCNT films at 9 different positions.

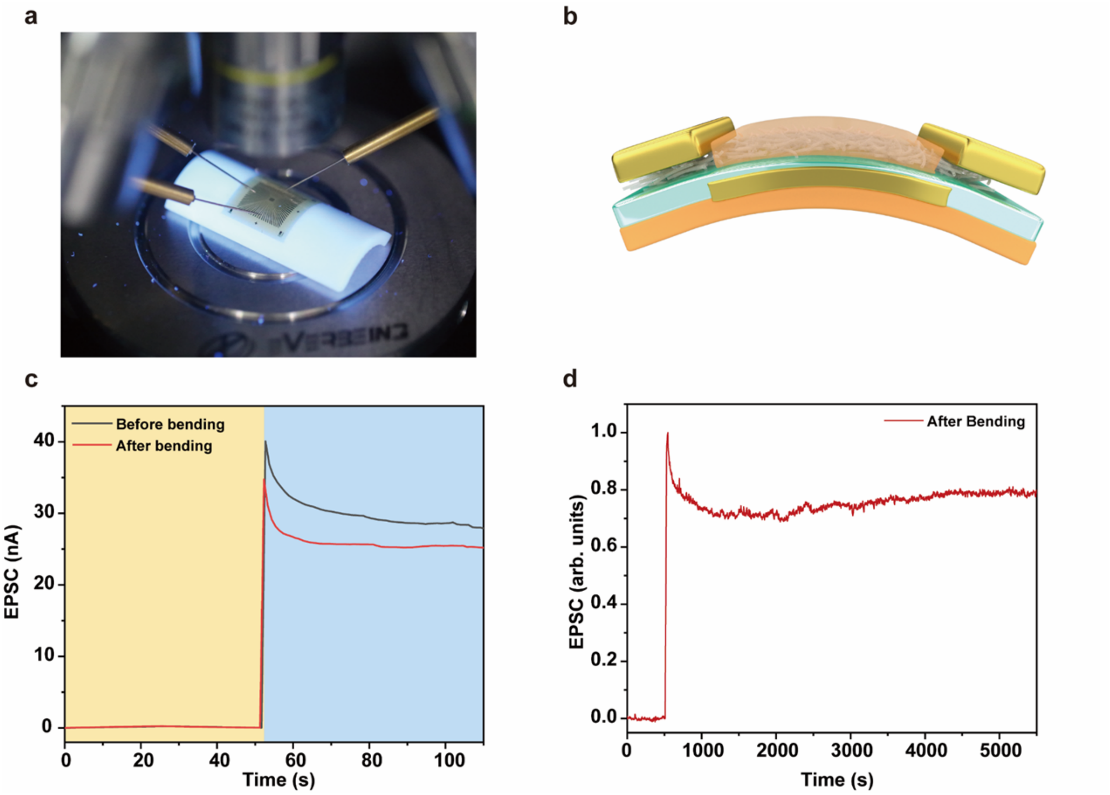

Supplementary Figure S24. (a) A photomicrograph and (b) illustration diagram of the flexibility test of the device. (c) Comparison of storage capacity before and after bending the device. (d) Long-term charge storage capacity of the device at a bent state. The light pulse has a width of 30 s, a P_{light} of 0.5 mW/cm^{-2} and a V_{DS} of 1V, respectively.

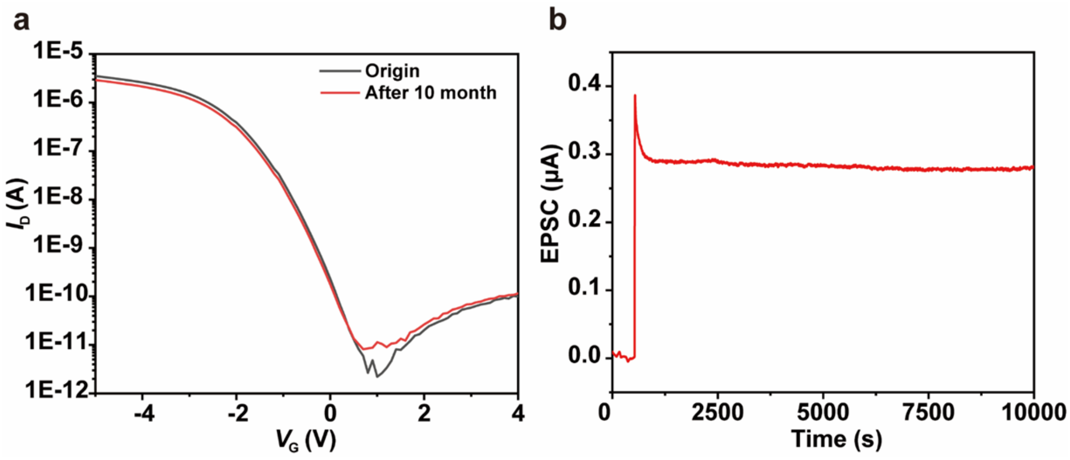

Supplementary Figure S25. Long-term stability of the device, (a) Transfer characteristic curves of the device stored approximately 10 months ago, (b) Storage capacity testing of the device after ten months. The light pulse has a width of 30 s, P_{light}=0.5 mW/cm^{-2}, V_{DS} = 1 V.

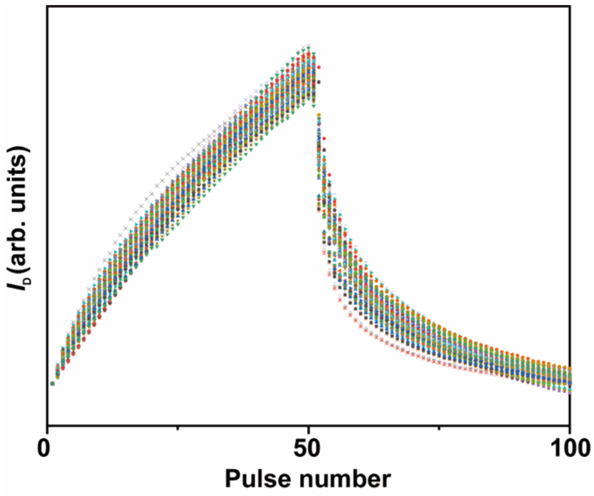

Supplementary Figure S26. Light-controlled LTP (light intensity 1 mW/cm^2, duration of 1 s, spaced 2 s apart) and V_G-controlled LTD (V_G=−1.5 V, duration of 1 s, spaced 1s apart) for 50 pulses under 77 K. V_{DS} = 1 V, a total of 100 SWCNT/ZnTPP phototransistors were tested.

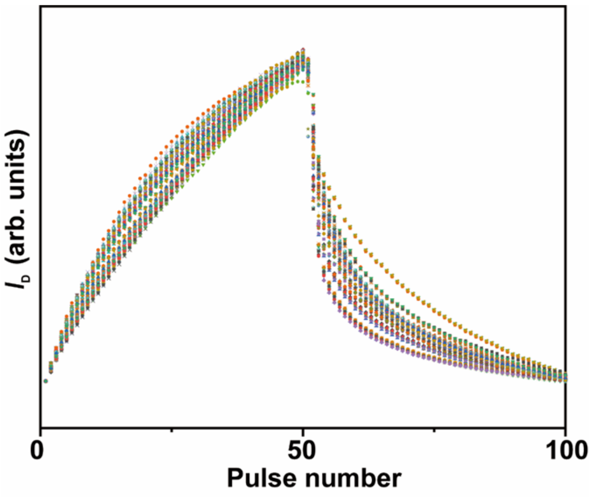

Supplementary Figure S27. Light-controlled LTP (light intensity 1 mW/cm^2, duration of 1 s, spaced 2 s apart) and V_G-controlled LTD (V_G=−1.5 V, duration of 1 s, spaced 1s apart) for 50 pulses under 300 K. V_{DS} = 1 V, a total of 100 SWCNT/ZnTPP phototransistors were tested.

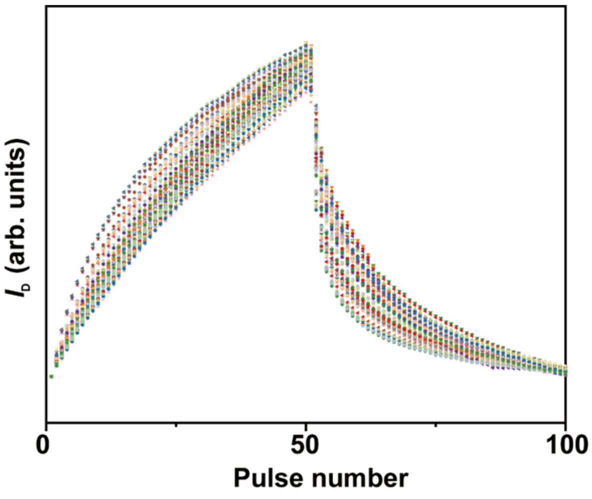

Supplementary Figure S28. Light-controlled LTP (light intensity 1 mW/cm^2, duration of 1 s, spaced 2 s apart) and V_G controlled LTD (V_G =−1.5 V, duration of 1 s, spaced 1s apart) for 50 pulses under 400 K. V_{DS} = 1 V, a total of 100 SWCNT/ZnTPP phototransistors were tested.

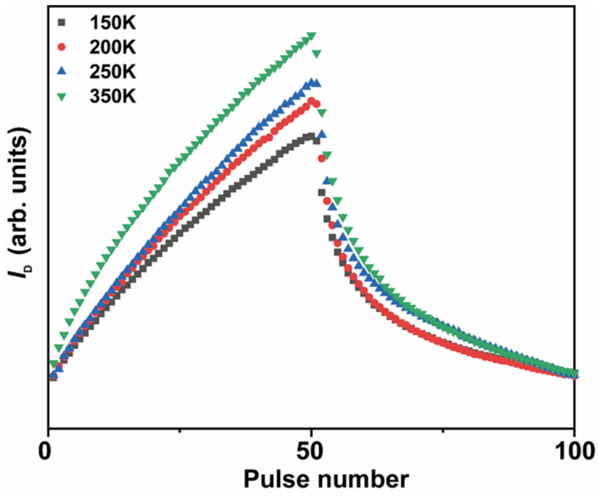

Supplementary Figure S29. Light-controlled LTP (light intensity 1 mW/cm^2, duration of 1 s, spaced 2 s apart) and V_G-controlled LTD (V_G=−1.5 V, duration of 1 s, spaced 1s apart) for 50 pulses under 150 K, 200 K, 250 K, 350 K, respectively. V_{DS} = 1 V.

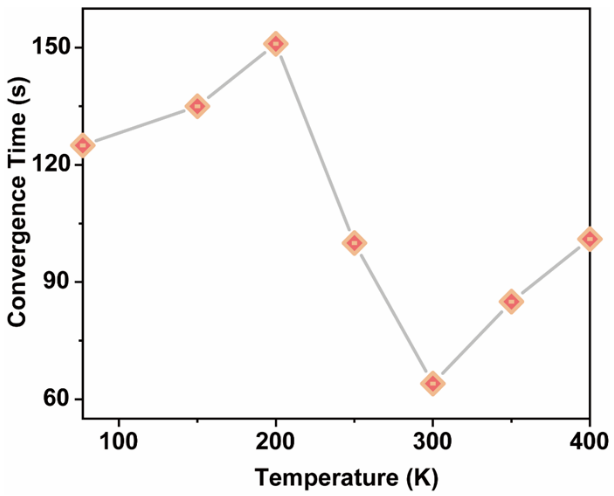

Supplementary Figure S30. Convergence time of training process at six different temperatures (77 K, 150 K, 200 K, 250 K, 300 K, 350 K, 400 K).

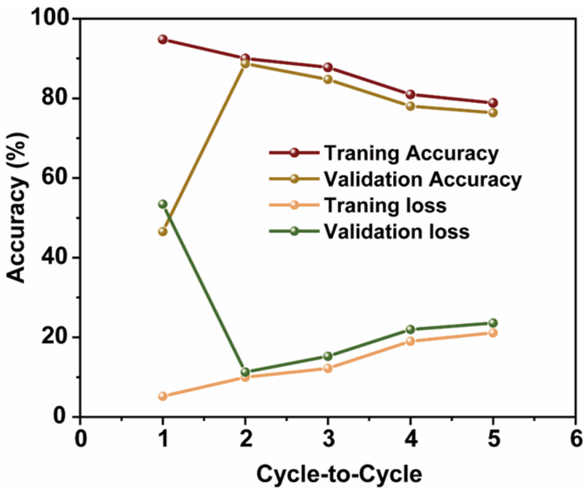

Supplementary Figure S31. The decease of training accuracy, loss and validation accuracy, loss within five cycles.

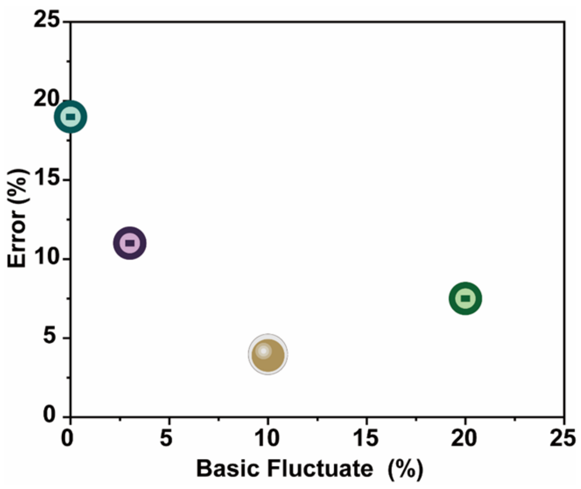

Supplementary Figure S32. The impact of setting initial weight values on recognition rate.

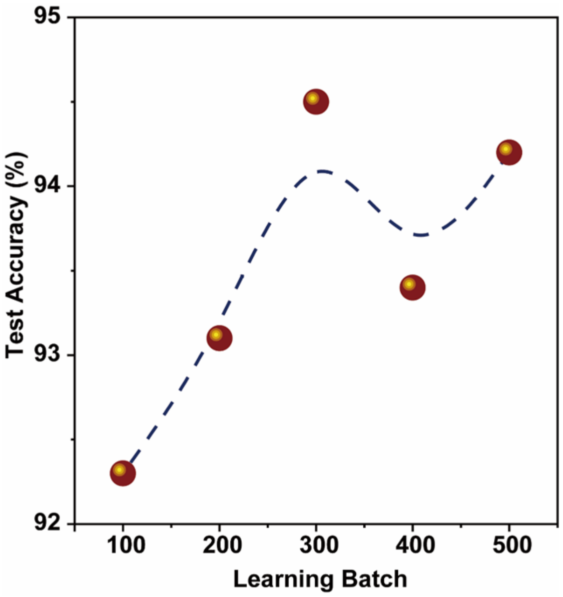

Supplementary Figure S33. Interference of learning batch during training on final instructions.

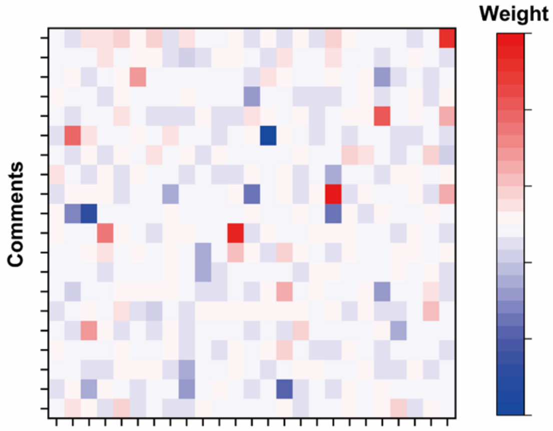

Supplementary Figure S34. The stable state of weight values in neural networks after training.

References:

1. Long, G. *et al.* Carbon nanotube-based flexible high-speed circuits with sub-nanosecond stage delays. *Nat. Commun.* **13**, 6734 (2022).
2. Zhang, H. *et al.* Wafer-Scale fabrication of ultrathin flexible electronic systems via capillary-assisted electrochemical delamination. *Adv. Mater.* **30**, 1805408 (2018).
3. Ding, J. *et al.* Enrichment of large-diameter semiconducting SWCNTs by polyfluorene extraction for high network density thin film transistors. *Nanoscale* **6**, 2328 (2014).
4. Wei, S. *et al.* Flexible quasi-2D perovskite/IGZO phototransistors for ultrasensitive and broadband photodetection. *Adv. Mater.* **32**, 1907527 (2020).
5. Choi, S. *et al.* Bio-Inspired complementary photoconductor by porphyrin-coated silicon nanowires. *Adv. Mater.* **23**, 3979–3983 (2011).
6. Huang, P.-Y. *et al.* Neuro-inspired optical sensor array for high-accuracy static image recognition and dynamic trace extraction. *Nat. Commun.* **14**, 6736 (2023).
7. Wang, Q. *et al.* Nonvolatile infrared memory in MoS_2 /PbS van der Waals heterostructures. *Sci. Adv.* **4**, eaap7916 (2018).
8. Chen, J., Chiu, Y., Li, Y., Chueh, C. & Chen, W. Nonvolatile perovskite-based photomemory with a multilevel memory behavior. *Adv. Mater.* **29**, 1702217 (2017).
9. Hao, J. *et al.* Low-energy room-temperature optical switching in mixed-dimensionality nanoscale perovskite heterojunctions. *Sci. Adv.* **7**, eabf1959 (2021).
10. Zhu, C. *et al.* Optical synaptic devices with ultra-low power consumption for neuromorphic computing. *Light. Sci. Appl.* **11**, 337 (2022).
11. Wang, Q. *et al.* Nonvolatile infrared memory in MoS_2/PbS van der Waals heterostructures. *Sci.*

Adv. **4**, eaap7916 (2018).

12. Wei, S. *et al.* Flexible quasi-2D perovskite/IGZO phototransistors for ultrasensitive and broadband photodetection. *Adv. Mater.* **32**, 1907527 (2020).

13. Liu, C. *et al.* Realizing the switching of optoelectronic memory and ultrafast detector in functionalized-black phosphorus/MoS_2 heterojunction. *Laser & Photonics Rev* **17**, 2200486 (2023).

14. Wang, Y. *et al.* Photonic synapses based on inorganic perovskite quantum dots for neuromorphic computing. *Adv. Mater.* **30**, 1802883 (2018).

15. Li, G. *et al.* Photo-induced non-volatile VO_2 phase transition for neuromorphic ultraviolet sensors. *Nat. Commun.* **13**, 1729 (2022).

16. Yang, B. *et al.* Bioinspired multifunctional organic transistors based on natural chlorophyll/organic semiconductors. *Adv. Mater.* **32**, 2001227 (2020).

17. Pradhan, B. *et al.* Ultrasensitive and ultrathin phototransistors and photonic synapses using perovskite quantum dots grown from graphene lattice. *Sci. Adv.* **6**, eaay5225 (2020).
