# Supplementary material for: Ultra-low power carbon nanotube/porphyrin synaptic arrays for persistent photoconductivity and neuromorphic computing
Source: Nat Commun. 2024 Jul 21;15:6147. doi: 10.1038/s41467-024-50490-y (PMC11271480; doi:10.1038/s41467-024-50490-y)
Supplement: Supplementary file 3 — Description of Additional Supplementary Information [file 41467_2024_50490_MOESM3_ESM.docx]

**Description of Additional Supplementary Files**

File Name: Supplementary Movie 1

Description: The simple demonstration showcased the edge computing potential of our chip. Combined with sensors and neural networks, it can handle part of the task of line tracking and recognition.
